# Confirmation of the Factor Structure and Reliability of the ‘Adult Eating Behavior Questionnaire’ in an Adolescent Sample

**DOI:** 10.3389/fpsyg.2019.01991

**Published:** 2019-10-04

**Authors:** Claudia Hunot-Alexander, Rebecca J. Beeken, William Goodman, Alison Fildes, Helen Croker, Clare Llewellyn, Silje Steinsbekk

**Affiliations:** ^1^Centro Universitario de Ciencias de la Salud, Departamento de Reproducción Humana, Crecimiento y Desarrollo Infantil, Instituto de Nutrición Humana, Universidad de Guadalajara, Guadalajara, Mexico; ^2^Leeds Institute of Health Sciences, Yorkshire Cancer Research University Academic Fellow, Leeds, United Kingdom; ^3^Department of Behavioural Science and Health, University College London, London, United Kingdom; ^4^School of Psychology, Faculty of Medicine and Health, University of Leeds, Leeds, United Kingdom; ^5^Department of Psychology, Norwegian University of Science and Technology, Trondheim, Norway

**Keywords:** appetite, appetitive traits, adolescents, eating, behavior, behavioral susceptibility theory

## Abstract

**Introduction:**

Appetitive traits, including Food Responsiveness, Enjoyment of Food, Satiety Responsiveness, Emotional Over- and Under-Eating, Food Fussiness and Slowness in Eating, have been captured across childhood using the Children’s Eating Behavior Questionnaire (CEBQ). The Adult Eating Behavior Questionnaire (AEBQ) has explored these traits in adults, but not adolescents. This study aimed to test the factor structure and reliability of the AEBQ in a sample of UK adolescents, and explore demographic differences.

**Materials and Methods:**

Confirmatory factor analysis (CFA) tested an 8-factor and a 7-factor AEBQ, based on valid, completed AEBQ responses (*n* = 913) from adolescents aged 11–18 recruited from four London secondary schools. Test–retest reliability was analyzed in a subsample (*n* = 106) 2-weeks later, and 492 participants completed the Dutch Eating Behavior Questionnaire (DEBQ) to assess convergent validity. Demographic differences were explored using a multiple indicator multiple cause (MIMIC) model.

**Results:**

The CFA revealed an adequate model fit for a 7-factor structure without Hunger [RMSEA = 0.038 (90% CI:0.035,0.041); CFI = 0.926, TLI = 0.916; and χ^2^(df = 595) = 8502.69, *p* < 0.001]. These seven subscales showed acceptable internal consistency (Cronbach’s α > 0.70). The ICC for the test–retest was above 0.70. Comparisons with the DEBQ supported the convergent validity of the AEBQ. Older age was associated with greater Food Responsiveness and Enjoyment of Food (all *p*-values < 0.005). Females reported higher levels of Emotional Over-Eating, Satiety Responsiveness, and Slowness in Eating than males (all *p*-values ≤ 0.003).

**Conclusion:**

This study supports the use of the 7-factor AEBQ as a reliable measure of appetitive traits in adolescents.

## Introduction

According to the Behavioral Susceptibility Theory (BST) of obesity (Carnell and Wardle, 2007; Llewellyn and Wardle, 2015), appetitive traits, such as Food Responsiveness [i.e., the tendency to eat (or eat more) in response to food cues such as the sight and smell of food] (Carnell and Wardle, 2008) and Satiety Responsiveness (i.e., the capacity to adjust eating in response to internal feelings of satiety or fullness) (Wardle et al., 2001; Carnell and Wardle, 2008) contribute to individual differences in energy intake, and ultimately weight status. A number of studies have demonstrated that Food Approach traits (e.g., Food Responsiveness, Emotional Over-Eating and Enjoyment of Food) are positively associated with patterns of overconsumption such as more frequent eating (Syrad et al., 2016) and predict excessive weight gain in childhood (van Jaarsveld et al., 2011, 2014; Steinsbekk and Wichstrøm, 2015). On the other hand Food Avoidance traits (e.g., Satiety Responsiveness, Emotional Under-Eating, Food Fussiness, and Slowness in Eating) are negatively associated with food intake patterns of undereating and weight cross-sectionally (Carnell and Wardle, 2008; Viana et al., 2008; Webber et al., 2009; Mallan et al., 2016; Syrad et al., 2016) and prospective weight gain (van Jaarsveld et al., 2011, 2014; Steinsbekk and Wichstrøm, 2015).

Research indicates that appetitive traits are moderately stable during childhood (Ashcroft et al., 2008; Steinsbekk and Wichstrøm, 2015; Steinsbekk et al., 2017), but also that Food Approach traits tend to increase while Food Avoidance traits tend to decrease with age (Ashcroft et al., 2008). This suggests that children tend to keep their relative position in the appetite hierarch over time, but on the whole the internal regulation of eating behaviors declines as children mature (i.e., intake is increasingly affected by external factors). During adolescence, young people are expected to take increasingly more responsibility for their daily habits, eating habits become unhealthier (Tăut et al., 2015), and these changes can last well into adulthood (Neumark-Sztainer et al., 2012; Larson et al., 2018). Thus, adolescence is the major formative transition period for one of the most decisive behaviors for adult health—healthy eating behavior.

However, little is known about the stability of appetitive traits throughout adolescence and into adulthood, partly due to a lack of valid and reliable age-appropriate measures that capture the full range of appetitive traits enshrined in the CEBQ. Psychometric measures capturing specific eating behaviors (a term sometimes used interchangeably with appetitive traits), such as Emotional Eating and Disinhibition (van Strein et al., 1986; Braet and van Strein, 1997; Vannucci et al., 2012), External Eating and Restraint (Wardle et al., 1992; Braet and van Strein, 1997; Caccialanza et al., 2004) and Eating in the Absence of Hunger (Tanofsky-Kraff et al., 2008) have been tested in adolescent samples, but these do not capture the full range of traits that have been explored in childhood (Wardle et al., 2001).

Adolescence is characterized by considerable physiological and psychological change (Eccles et al., 1993), and measures validated in child samples may not be appropriate for adolescents. While parent-reported measures of eating behavior have been applied in preadolescent samples (Wardle et al., 2001; Caccialanza et al., 2004), information provided by parents does not always mirror that of self-report (Achenbach et al., 1987). Parents and adolescents are discordant in reporting dietary intake (Northstone et al., 2013) and eating disorder symptoms (Swanson et al., 2014). It is therefore possible that adolescents and their parents will similarly differ in their report of appetitive traits.

A self-report adult version of the parent-report CEBQ, the most extensively used measure of appetitive traits in children (Wardle et al., 2001; Croker et al., 2011), was recently developed to capture the specific appetitive traits measured by the CEBQ in adulthood (Hunot et al., 2016). The AEBQ characterizes eight appetitive traits: Hunger; Food Responsiveness; Emotional Over-Eating; Enjoyment of Food; Satiety Responsiveness; Emotional Under-Eating; and Slowness in Eating. Initial testing of the AEBQ in a sample of 954 British adults demonstrated the eight AEBQ scales had good internal reliability (α: 0.76–0.88) and test–retest reliability (ICCs: 0.73–0.91) (Hunot et al., 2016). Reliability was also demonstrated in a study testing the AEBQ in a sample of 998 young Australian adults (mean age 24.32 years [*SD* = 8.32]) (Mallan et al., 2017). Together with the CEBQ, the AEBQ enables the longitudinal tracking of appetitive traits from childhood into adulthood. However, it has not been used with adolescents, and so it is unclear whether the self-report AEBQ is appropriate for use in adolescent samples.

In addition, the AEBQ has not been validated against other self-reported measures of appetitive traits. The DEBQ (van Strein et al., 1986) is one of the most widely used measures of appetitive traits in adolescents and adults. The Emotional eating subscale of the DEBQ is similar to the Emotional Over-Eating subscale of the AEBQ, and External Eating (DEBQ) is thought to correspond to the Food Responsiveness subscale (AEBQ). On the other hand, the DEBQ Restrained Eating subscale, is characterized by undereating and thus likely to be inversely related to the AEBQ Food Approach scales, and positively related to the AEBQ Food Avoidance subscales (i.e., Satiety Responsiveness, Food Fussiness, Slowness in Eating). Comparing scores on the AEBQ with scores on the relevant scales of the DEBQ would therefore give an indication of the convergent validity of the AEBQ.

The present research aimed to (i) confirm the factor structure of the AEBQ in a large sample of UK adolescents aged 11–18 years; (ii) examine both internal and test–retest reliability of the AEBQ in this sample; (iii) assess convergent validity of the AEBQ through comparisons with the DEBQ; and (iv) explore demographic differences in appetitive traits. Childhood studies indicate that Food Approach traits increase, whereas Food Avoidance traits decrease with age from early to late childhood (Ashcroft et al., 2008), so we hypothesized that older adolescents would have higher scores for the Food Approach subscales of the AEBQ, and lower scores for Food Avoidance subscales. Furthermore, disordered eating, which typically emerges in adolescence, is more prevalent in females (Neumark-Sztainer et al., 2002), and adolescent females have been shown to display more emotional eating than males (Lu et al., 2016). We therefore hypothesized that females would score higher on both Emotional Over- and Under-Eating compared to males. Differences by ethnicity and maternal education are also investigated, but these analyses were exploratory and not hypothesis-driven.

## Materials and Methods

### Participants and Procedure

Eighteen Secondary schools (Years 7 through 13, pupils aged 11–18) in the London area were contacted by mail in 2013–2014 and invited to participate. Four (22.2%) schools consented to take part and were sent study information sheets to be distributed to parents, along with parent opt-out consent forms for students under 16 years of age (this was not required for those 16 years and older). Students aged 16 and older, and students under the age of 16 whose parents had not opted out, were provided with oral and written information about the study on the day of assessment. Interested students were asked to sign consent forms for completion of the questionnaire and provision of demographic data. The only exclusion criterion was the student not speaking English. Ethical approval was obtained from University College London Research Ethics Committee (4378/001).

To examine test–retest reliability, a subsample of students (Subsample I) from 4 randomly selected classes from one school were also asked to complete the AEBQ a second time, 2 weeks after baseline. Another subsample (Subsample II) (all participating students from two out of the four schools) also completed the DEBQ (van Strein et al., 1986).

### Measures

#### The Adult Eating Behavior Questionnaire

The AEBQ (Hunot et al., 2016) consists of 35 items measured along a 5-point Likert scale (1 = “strongly disagree”; 5 = “strongly agree”), constituting eight subscales, conceptually grouped into Food Approach and Food Avoidance scales, based on them being either positively or negatively related to weight (Wardle et al., 2001; Hunot et al., 2016). Four Food Approach scales are comprised of: Hunger (five items, e.g., “I often feel so hungry that I have to eat something right away”); Food Responsiveness (four items, e.g., “When I see or smell food that I like, it makes me want to eat”); Emotional Over-Eating (five items, e.g., “I eat more when I’m anxious”); and Enjoyment of Food (three items, e.g., “I love food”). Four Food Avoidance scales include: Satiety Responsiveness (four items, e.g., “I often leave food on my plate at the end of a meal”); Emotional Under-Eating (five items, e.g., “I eat less when I’m worried”); Food Fussiness (five items, e.g., “I refuse new foods at first”) and Slowness in Eating (i.e., eating rate, four items, e.g., “I am often last at finishing a meal”). Before the AEBQ was given to the adolescents, Think Aloud interviews (Fox et al., 2011) were carried out with six adolescents, two 12 year olds, one 14 year old and three 15 year olds, to assess the overall readability and comprehension of the questionnaire. Participants understood the questionnaire and a Flesch Reading Ease assessment gave the AEBQ an easy to read score (81.8) (Readability Formulas, 2018).

#### Dutch Eating Behavior Questionnaire

The DEBQ (van Strein et al., 1986), a validated measure of eating behavior in adults, was used to assess convergent validity of the AEBQ (Wardle, 1987; Lluch et al., 1996; Cebolla et al., 2014). The DEBQ consists of 33 items capturing the following eating behavior dimensions: Emotional Eating (13 items, e.g., “Desire to eat when things go wrong”), External Eating (10 items, e.g., “Desire to eat when watching others eat”) and Restrained Eating (10 items, e.g., “Eat less to avoid weight gain”), measured along a five-point Likert scale (1 = never, 5 = very often).

### Sociodemographics

Participants reported their gender, age, ethnicity (categorized as white and non-white), and paternal and maternal level of education (attended college: yes, no, or don’t know) as a proxy for socio-economic status (Vereecken et al., 2004; Northstone and Emmett, 2005; Jones et al., 2010; Cribb et al., 2011).

### Statistical Analyses

To explore whether Subsample I and II differed from the whole sample in terms of their baseline characteristics and AEBQ scores, Chi-squared tests were used for categorical and Student *t*-tests for continuous variables. Pearson’s correlations were carried out to show the associations between appetitive traits, for all traits. For the CFA, a robust maximum likelihood estimator was applied, and the following indicators were used to assess the goodness of fit of the model: The CFI, the TLI and the RMSEA. Values close to 0.95 for CFI and TLI are considered indications of excellent fit, whereas RMSEA values of less than 0.05 indicates an adequate fit, with the lower-bound confidence interval closest to zero (0) and the higher-bound confidence interval less than 0.08 (Marsh et al., 2004, 2009). Both the original development study for the AEBQ (Hunot et al., 2016), and a recent validation in an Australian sample (Mallan et al., 2017), suggested the Hunger scale could be omitted from the AEBQ. We therefore compared the original factor structure with a 7-factor solution (omitting ‘Hunger’). AIC and BIC, which are adjusted comparative fit indices were used to compare the models (Dugard et al., 2010). Models with smaller AIC and BIC are usually considered more parsimonious (Kline, 2016).

Cronbach’s alpha was used to test internal reliability for each appetitive trait and ICC using a Cronbach’s alpha model of greater than or equal to 0.70 assessed test–retest reliability, 2 weeks after the first questionnaire was answered (McGraw and Wong, 1996; Field, 2013; Streiner and Norman, 2015). Omega coefficients were calculated to eliminate potential errors in the estimation of reliability.

Because the CEBQ has been validated in 11–13 years olds, our sample was split into two age categories, an 11–13 years category and a 14–18 years category. To check the performance of the questionnaire in both of these age groups, we re-ran the reliability analysis to produce Cronbach alphas and Omega coefficients for the scales within the 11–13 year-olds and the 14–18 year-olds. We also tested for measurement invariance across three conditions: configural invariance, metric invariance and scalar invariance, to ensure that the AEBQ items had the same meaning across both age groups. Firstly, the 7-factor model was run on both groups separately with the chi-squared test, RMSEA, CFI and IFI used to assess the goodness of the model fit (Costa et al., 2015). Configural invariance identifies whether the factor structure is similar across groups; metric invariance constrains the factor loadings to be consistent across groups and scalar invariance constrains the intercepts to be equal across both groups. These invariance tests were run using multi-group CFA with the model fit indices (chi-squared test, RMSEA, CFI, and IFI) used to assess for invariance.

Internal consistency for each of the DEBQ subscales from our sample (Subsample II) were also calculated. Pearson’s correlations between the AEBQ and DEBQ subscales, were conducted to test convergent validity.

To assess whether age, gender, ethnicity, maternal education and paternal education (College education yes/no, ‘don’t know,’ where ‘don’t know’ was treated as missing) were associated with each appetitive trait, and to take into account the possible association between the subscales of the AEBQ, we ran a CFA with covariates method (MIMIC model) in which all subscales of the AEBQ were defined as latent variables, and the covariates as independent variables (Joreskog and Goldberger, 1975).

CFA was conducted in Mplus version 7.0 (Muthén and Muthén, 2007). Invariance testing and MIMIC were run in SPSS AMOS 24.0. All other analyses were conducted in SPSS 24.0. To adjust for multiple analysis, *p* levels were set at <0.01 (Bland and Altman, 1995).

## Results

A total of 1160 students consented to participate, and 972 (83.8%) adolescents completed the AEBQ at baseline. Of these, 59 (6.5%) responses were eliminated due to extreme response sets (a majority of extreme answers on the response format, e.g., ‘strongly disagree’ and ‘strongly agree’). Extreme response sets can indicate general indifference to the questionnaire, so data from these participants were considered unreliable (Allison and Baskin, 2009; Field, 2013). This left 913 responses for analysis. A subsample of 106 students (Subsample I) completed the AEBQ for a second time to allow for test–retest reliability to be calculated. A second subsample of 492 participants (Subsample II; all participating students from 2/4 schools) also completed the DEBQ (van Strein et al., 1986) to assess convergent validity.

Baseline characteristics and baseline AEBQ scores for Subsamples I and II were compared to the full sample (Table 1). There was a higher proportion of 14–18 year olds within Subsample I compared to the full sample [χ^2^(1) = 16.814, *p* < 0.001]. There was a higher proportion of 14–18 year olds within Subsample II compared the full sample [χ^2^(1) = 34.532, *p* < 0.001] (Table 1).

**TABLE 1:** Sample characteristics at baseline.

	**Total sample (*n* = 913)**	**Subsample I: Test–retest (*n* = 106)**	**Group difference between total Sample and Subsample I (test statistic, *p*)**	**Subsample II: Completed AEBQ and DEBQ (*n* = 450)^b^**	**Group difference between total sample and Subsample II (test statistic, *p*)**
Age (years, mean ± SD)	14.35 ± 1.72	13.57 ± 1.47	*t*(911) = 4.416, *p* < 0.001	14.17 ± 0.89	*t*(911) = 1.680, *p* = 0.093
Age categories	11–18 y	11–18 y		13–16 y	
11–13	304 (33.3%)	54 (50.9%)	χ^2^(1) = 16.814, *p* < 0.001	108 (24%)^c^	χ^2^(1) = 34.532, *p* < 0.001
14–18	609 (66.7%)	52 (49.1%)		342 (76%)^d^	
Gender					
Male	450 (49.3%)	49 (46.2%)	χ^2^(1) = 0.450, *p* = 0.502	240 (53.5%)	χ^2^(1) = 5.810, *p* = 0.016
Female	463 (50.7%)	57 (53.8%)		210 (46.7%)	
Adolescent ethnicity^a^	(*n* = 900)	(*n* = 104)		(*n* = 439)	
White	309 (34.3%)	45 (42.9%)	χ^2^(1) = 3.831, *p* = 0.063	136 (31.0%)	χ^2^(1) = 4.276, *p* = 0.042
Non-white	591 (65.7%)	60 (57.1%)		303 (69.0%)	
Maternal education^a^	(*n* = 907)	(*n* = 104)			
College/university education					
Yes	337 (37.3%)	37 (35.2%)	χ^2^(2) = 3.477, *p* = 0.176	162 (36.0%)	χ^2^(2) = 1.844, *p* = 0.398
No	259 (28. 6%)	24 (22.9%)		124 (27.6%)	
Don’t know	311 (34.3%)	44 (41.9%)		164 (36.4%)	
Paternal education^a^	(*n* = 906)	(*n* = 103)		(*n* = 449)	
College/university education					
Yes	333 (36.8%)	30 (28.6%)	χ^2^(2) = 9.172, *p* = 0.010	176 (39.2%)	χ^2^(2) = 6.227, *p* = 0.044
No	213 (23.5%)	19 (18.1%)		90 (20.0%)	
Don’t know	360 (39.7%)	56 (53.3%)		183 (40.8%)	

*y, years old. ^*a*^Ethnicity and parental education is reported by the adolescent. ^*b*^To assess convergent validity (*n* = 450; 42/492 eliminated due to extreme response set). ^*c*^Only 13 year olds are included. ^*d*^Only 14–16 year olds are included.*

### Confirmatory Factor Analysis and Exploratory Structural Equation Modeling

CFA of the original factor structure (i.e., eight subscales) revealed an adequate model fit [RMSEA = 0.038 (90% CI:0.035,0.041); CFI = 0.912, TLI = 0.902; and χ^2^(df = 595) = 8502.69, *p* < 0.001], which was slightly improved by omitting the ‘Hunger’ subscale [RMSEA = 0.038 (90% CI:0.035,0.041); CFI = 0.926, TLI = 0.916; and χ^2^(df = 595) = 8502.69, *p* < 0.001]. The lower AIC and BIC values of the 7-factor solution (AIC 7-factors = 74221.375 vs. AIC 8-factors = 87081.142; BIC 7-factors = 74756.033 vs. BIC 8-factors = 87721.768) indicates that the model without Hunger is better, given that models with the lowest AIC‘s and BIC’s indicate the best fit (Dugard et al., 2010). The factor loadings and 95% confidence intervals of the CFA are displayed in Table 2.

**TABLE 2:** AEBQ: Internal and external reliability measures and factor loadings.

	**Items**	**Internal reliability Cronbach’s alpha and omega coefficient Total sample (*n* = 913)**	**Test–retest reliability ICC (*n* = 105)**	**Standardized factor loadings CFA – 8 factors Total sample (*n* = 913)**	**Standardized factor loadings CFA – 7 factors Total sample (*n* = 913)**

		**α (ω)**	**α**	**λ (95% CI)**	**λ (95% CI)**
H	Q6	0.662 (0.671)	0.861	0.510 (0.444,0.576)	a
	Q28			0.596 (0.534,0.659)	a
	Q32			0.660 (0.600,0.720)	a
	Q34			0.426 (0.350,0.502)	a
	Q9			0.488 (0.419,0.556)	a
FR	Q22	0.709 (0.710)	0.884	0.664 (0.617,0.710)	0.650 (0.599,0.700)
	Q17			0.654 (0.604,0.705)	0.692 (0.641,0.743)
	Q13			0.584 (0.531,0.637)	0.571 (0.513,0.630)
	Q33			0.552 (0.491,0.613)	0.540 (0.472,0.608)
EOE	Q10	0.800 (0.804)	0.783	0.779 (0.736,0.922)	0.778 (0.735,0.821)
	Q8			0.749 (0.699,0.799)	0.754 (0.705,0.804)
	Q16			0.621 (0.555,0.687)	0.618 (0.552,0.685)
	Q5			0.706 (0.656,0.756)	0.705 (0.655,0.756)
	Q21			0.487 (0.409,0.564)	0.484 (0.406,0.562)
EF	Q3	0.800 (0.810)	0.919	0.797 (0.752,0.841)	0.798 (0.754,0.843)
	Q1			0.789 (0.744,0.833)	0.790 (0.745,0.834)
	Q4			0.710 (0.659, 761)	0.707 (0.656,0.758)
SR	Q31	0.740 (0.745)	0.860	0.710 (0.655, 766)	0.711 (0.655,0.766)
	Q30			0.509 (0.442,0.577)	0.509 (0.441,0.577)
	Q11			0.652 (0.595,0.709)	0.652 (0.595,0.709)
	Q23			0.707 (0.650,0.764)	0.706 (0.650,0.763)
EUE	Q27	0.792 (0.797)	0.814	0.764 (0.710, 817)	0.763 (0.709,0.816)
	Q15			0.601 (0.530,0.672)	0.601 (0.531,0.672)
	Q35			0.561 (0.489,0.633)	0.560 (0.488,0.632)
	Q20			0.764 (0.716, 811)	0.766 (0.718,0.813)
	Q18			0.592 (0.522,0.622)	0.592 (0.522,0.622)
FF	Q7	0.781 (0.788)	0.887	0.508 (0.437,0.580)	0.509 (0.437,0.580)
	Q19^∗^			0.801 (0.751,0.852)	0.801 (0.751,0.852)
	Q2			0.443 (0.369,0.517)	0.443 (0.369,0.517)
	Q12^∗^			0.770 (0.720,0.820)	0.769 (0.720,0.819)
	Q24^∗^			0.675 (0.621,0.729)	0.675 (0.622,0.729)
SE	Q29	0.756 (0.764)	0.875	0.809 (0.760,0.859)	0.809 (0.759,0.858)
	Q25			0.750 (0.698,0.802)	0.750 (0.697,0.802)
	Q14^∗^			0.546 (0.471, 621)	0.549 (0.475,0.623)
	Q26			0.540 (0.470, 611)	0.540 (0.469,0.610)

*^∗^Items were reversed for scoring. H, Hunger; FR, Food Responsiveness; EOE, Emotional Over-Eating; EF, Enjoyment of Food; SR, Satiety Responsiveness; EUE, Emotional Under-Eating; FF, Food Fussiness; SE, Slowness in Eating; ICC, Intra-Class Correlation coefficient; CFA, Confirmatory Factor Analysis; α, Cronbach’s alpha; λ, Factor loading; CI, Confidence interval; a, the Hunger subscale was not included in the model tested, thus factor loadings are not estimated; ω, omega coefficient.*

The results of the 7-factor model for both age groups are presented in Table 3. The chi-squared test was significant for both groups, indicating poor model fit, whereas the RMSEA indicated good fit for both the 11–13 year olds (0.053) and 14–18 year olds (0.045). The CFI and IFI differentiated more between the groups indicating a better fit for the 14–18 year olds (CFI = 0.908; and IFI = 0.909). The results for the multi-group CFA can be found in Table 4. These suggest that there is non-invariance in the models between the two age groups, based upon the significant chi-squared tests and the CFI and IFI below 0.9. However, the RMSEA indicated good model fit and remained consistent (change of 0.01) between the unconstrained and constrained models, therefore we continued with subsequent analyses.

**TABLE 3:** Model fit indices for a 7-factor model of both age groups.

**Model fit indices**	**Age**	
	
	**11–13**	**14–18**
χ^2^ (degrees of freedom)	989.67 (532)	1184.18 (532)
χ^2^ *p*-value	<0.001	<0.001
RMSEA	0.053	0.045
CFI	0.840	0.908
IFI	0.845	0.909

*RMSEA, Root mean square error of approximation; CFI, Comparative fit index; IFI, Incremental fit index.*

**TABLE 4:** Model fit indices for the unconstrained and constrained analyses of invariance by age.

	**χ^2^ (degrees of freedom)**	***P*-value**	**RMSEA (90% CI)**	**CFI**	**IFI**
Age					
Unconstrained (Configural)	2174.30 (1064)	<0.001	0.034 (0.032–0.036)	0.888	0.891
Partially constrained (Metric)	2241.37 (1091)	<0.001	0.034 (0.032–0.036)	0.884	0.886
Difference (Metric vs. Configural)	67.07 (27)	<0.001			
Constrained (Scalar)	2349.84 (1126)	<0.001	0.035 (0.033–0.037)	0.877	0.879
Difference (Scalar vs. Metric)	108.48 (35)	<0.001			

*RMSEA, Root mean square error of approximation; CFI, Comparative fit index; IFI, Incremental fit index.*

### Internal and Test–Retest Reliability

The internal reliability estimates and the test–retest results are presented in Table 2. Cronbach’s alpha was >0.70 for all but one (Hunger) of the appetitive traits. The ICCs for the test–retest results were also above 0.70, suggesting the AEBQ has acceptable test–retest reliability in this sample. Both Cronbach’s alpha and omega coefficient values were very similar.

Reliability analysis for the two age groups (11–13 years olds and 14–18 years olds) showed that all subscales were reliable for both except for Hunger in the 11–13 year-olds (α = 0.579), and the 14–18 year-olds (α = 0.684). Reliability was slightly below 0.70 for Satiety Responsiveness in the 11–13 year-olds (α = 0.694) (Table 5).

**TABLE 5:** Internal reliability of the sample by age categories.

**Appetitive traits**	**Internal reliability Cronbach’s alpha and omega coefficient**	
	
	**11–13 years (*n* = 304) (ω)**	**14–18 years (*n* = 609) (ω)**
Hunger	0.579 (0.603)	0.684 (0.690)
Food Responsiveness	0.707 (0.707)	0.700 (0.702)
Emotional Over-Eating	0.779 (0.782)	0.806 (0.811)
Enjoyment of Food	0.715 (0.737)	0.824 (0.831)
Satiety Responsiveness	0.694 (0.695)	0.755 (0.782)
Emotional Under-Eating	0.767 (0.710)	0.804 (0.809)
Food Fussiness	0.782 (0.786)	0.775 (0.786)
Slowness in Eating	0.710 (0.720)	0.773 (0.781)

*ω, omega coefficient.*

### Associations Between Appetitive Traits

Table 6 shows the means and standard deviations for each subscale of the AEBQ, as well as correlations between AEBQ subscales. As expected the Food Approach subscales were positively inter-correlated and were generally negatively correlated with the Food Avoidance subscales, except for Hunger. Emotional Under- Eating was unexpectedly positively correlated to Hunger, although the size of the correlation was small. Emotional Over-Eating was not, however, significantly correlated to any Food Avoidance subscales (Table 6).

**TABLE 6:** Pearson’s correlations between the eight AEBQ subscales and three DEBQ subscales in a sample of adolescents (*n* = 972).

**Subscales**	**Mean ± SD**	**H**	**FR**	**EOE**	**EF**	**SR**	**EUE**	**FF**	**SE**
**AEBQ Food Approach subscales**									
Hunger	2.9 ± 0.7	1	0.646^∗^	0.455^∗^	0.442^∗^	−118^∗^	0.077	−0.096^∗^	–0.050
Food responsiveness	3.1 ± 0.8		1	0.433^∗^	0.600^∗^	−0.262^∗^	–0.010	−0.182^∗^	−0.192^∗^
Emotional over-eating	2.4 ± 0.8			1	0.263^∗^	–0.019	–0.057	–0.055	0.009
Enjoyment of food	4.0 ± 0.7				1	−0.370^∗^	–0.058	−0.308^∗^	−0.195^∗^
**AEBQ Food Avoidance subscales**									
Satiety responsiveness	2.8 ± 0.9					1	0.257^∗^	0.317^∗^	0.468^∗^
Emotional under-eating	2.9 ± 0.8						1	0.054	0.146^∗^
Food fussiness	2.7 ± 0.8							1	0.138^∗^
Slowness in eating	2.8 ± 8								1
**DEBQ**									
External Eating	3.1 ± 0.7	0.558^∗^	0.662^∗^	0.262^∗^	0.447^∗^	−0.201^∗^	0.016	−0.194^∗^	–0.110
Emotional Eating	2.2 ± 0.8	0.424^∗^	0.333^∗^	0.564^∗^	0.103	–0.031	–0.033	–0.016	0.008
Restrained eating	2.4 ± 0.9	–0.100	−0.183^∗^	–0.026	−0.271^∗^	0.327^∗^	−0.198^∗^	−0.147^∗^	0.190^∗^

*H, Hunger; FR, Food Responsiveness; EOE, Emotional Over-Eating; EF, Enjoyment of Food; SR, Satiety Responsiveness; EUE, Emotional Under-Eating; FF, Food Fussiness; SE, Slowness in Eating; AEBQ, Adult Eating Behavior Questionnaire; DEBQ, Dutch Eating Behavior Questionnaire. ^∗^Correlation is significant at the 0.01 level (two-tailed).*

### Convergent Validity

Internal consistency of the DEBQ subscales in our adolescent sample were as follows; Emotional eating: α = 0.80, External eating: α = 0.91 and Restrained Eating: α = 0.88, which is comparable to numbers reported in the development of the DEBQ (van Strein et al., 1986). Table 6 presents the correlations between the AEBQ and DEBQ scales, as well as means and standard deviations for each scale of the DEBQ. The AEBQ Emotional Over-Eating subscale was positively associated with the DEBQ Emotional Eating subscale (*r* = 0.56, *p* < 0.001), and the AEBQ Food Responsiveness subscale correlated with the comparable DEBQ External Eating subscale (*r* = 0.66, *p* < 0.001), indicating good convergent validity of the AEBQ against the DEBQ for similar constructs. The Restrained Eating subscale of the DEBQ was negatively associated with the AEBQ Food Approach subscales, and positively associated with the AEBQ Food Avoidance subscales, with the exception of Food Fussiness and Emotional Under-Eating, which were both negatively associated with restrained eating (*r* = −0.15, *p* < 0.001, and *r* = −0.20, *p* < 0.001 respectively).

### Sociodemographic Differences in Appetitive Traits

Neither maternal nor paternal education were associated with any of the traits; thus these results are not included within Table 7. MIMIC model regression estimates revealed that age was positively associated with Food Responsiveness and Enjoyment of Food; older adolescents were more likely to report higher levels of Food Responsiveness and Enjoyment of Food, indicating that adolescents become more appetitive as they get older (all *p* < 0.001). Thus, for example, an increase in 1 year for age, resulted in an increase of.10 points for Food Responsiveness (*p* < 0.001), and 0.08 points for Enjoyment of Food. In contrast no Food Avoidance traits were associated with age (Table 7).

**TABLE 7:** MIMIC model regression estimates for age, gender and ethnicity for all AEBQ subscales (*n* = 376).

	**Age**			**Gender**			**Ethnicity**		
			
**AEBQ scales**	**B (*SE*)**	**95% CI**	***p*-value**	**B (*SE*)**	**95% CI**	***p*-value**	**B (*SE*)**	**95% CI**	***p*-value**
Hunger	−0.01(0.03)	−0.07–0.06	0.487	0.12 (0.08)	−0.03–0.46	0.085	0.21 (0.12)	−0.00–0.52	0.052
Food responsiveness	0.10 (0.03)	0.05–0.17	< 0.001	0.11(0.08)	−0.09–0.31	0.312	0.01 (0.09)	−0.25–0.18	0.743
Emotional over-eating	0.02 (0.03)	−0.04–0.08	0.515	0.43 (0.10)	0.21–0.62	0.003	−0.20(0.10)	−0.40–0.02	0.073
Enjoyment of food	0.08 (0.02)	0.04–0.12	0.001	0.04 (0.06)	−0.09–0.18	0.564	0.08 (0.07)	−0.07–0.24	0.292
Satiety responsiveness	−0.05(0.02)	−0.10–0.01	0.087	0.48 (0.09)	0.28–0.69	0.002	0.04 (0.09)	−0.16–0.23	0.695
Emotional Under-eating	0.02 (0.03)	−0.04–0.08	0.477	0.14 (0.08)	−0.05–0.36	0.136	0.05 (0.09)	−0.17–0.24	0.695
Food fussiness	−0.03(0.02)	−0.07–0.01	0.126	0.05 (0.06)	−0.08–0.19	0.427	−0.03(0.06)	−0.17–0.12	0.724
Slowness in eating	−0.01(0.02)	−0.06–0.03	0.642	0.34 (0.07)	0.17–0.51	0.002	−0.02(0.07)	−0.19–0.13	0.763

*Age (years): Mean (*SD*); 14.26 (1.71). Gender: Male = 1 and Female = 2. Ethnicity: White = 1 and Non-white = 2. B coefficient, unstandardized values of β. SE, standard error.*

Gender was also associated with some Food Approach traits. Females were more likely to report higher scores for Emotional Over-Eating (*p* = 0.003); but not for Hunger, Food Responsiveness or Enjoyment of Food (all *p* > 0.05). The Food Avoidance traits Satiety Responsiveness and Slowness in Eating were also associated with gender; with females also scoring more highly for these traits (both *p* = 0.002). There was no association between gender and Food Fussiness (*p* = 0.427) (Table 7).

Neither ethnicity nor maternal education were associated with any of the Food Avoidance or Food Approach traits (Table 7).

## Discussion

The present study aimed to examine the reliability of the AEBQ in a large sample of UK adolescents. Our findings support the use of the 7-factor AEBQ as a reliable measure of appetitive traits in adolescents and highlight demographic differences in some of the traits.

The finding that the reliability and validity of the AEBQ may be improved by removing the Hunger subscale is in accordance with the results from the development of the AEBQ paper (Hunot et al., 2016) and the recent validation of the scale in younger Australian adults (Mallan et al., 2017). Together these results suggest using the AEBQ without the Hunger subscale may be a more appropriate option for future studies. The Hunger subscale captures physical aspects of hunger, which people may find difficult to assess due to issues with eating regulation (Karlsson et al., 2000). Factors such as adiposity, physical activity, eating disorders, and dieting behaviors could also contribute to individual differences in interpretations or perception of hunger.

Because this is the first study to examine the reliability of the AEBQ in adolescents, direct comparison with earlier studies is not possible. Notably, one previous attempt to translate the CEBQ into a self-report instrument showed low validity in a multi-ethnic sample of 13 year-old Malaysian adolescents (Loh et al., 2013). The present findings indicate that the AEBQ, on the other hand, is a valid and reliable measure of appetitive traits in adolescents ranging from 11 to 18 years of age, although replications in other samples, and for other forms of validity, are needed to support our findings. The convergent validity of the questionnaire is supported by significant associations between specific subscales of the AEBQ and their related counterparts in the DEBQ. Individuals who scored higher on the Food Responsiveness scales of the AEBQ were more likely to report higher External Eating on the DEBQ. Similarly, adolescents who scored higher on the AEBQ Emotional Over-Eating scale were also more likely to show higher levels of Emotional Eating on the DEBQ. Adolescents who reported higher Food Fussiness or Emotional Under-Eating on the AEBQ also reported less Restrained Eating on the DEBQ, however, the size of these associations was small.

We found that older adolescents were more susceptible to external food cues; and enjoyed food more. These results are consistent with findings from the CEBQ (Wardle et al., 2001) and reflect existing research in children. Although appetitive traits are moderately stable across childhood (Ashcroft et al., 2008), Food Responsiveness and Enjoyment of Food have been found to increase linearly with age (Wardle et al., 2001; Viana et al., 2008). This also mirrors research reporting that children’s self-regulation of eating decreases as they get older (Johnson and Taylor-Holloway, 2006). It is not clear if these changes are biologically or environmentally driven (e.g., older children and adolescents spend more time away from home and have more autonomy over food choices and are therefore more exposed to food cues, which might trigger and reinforce externally driven or food-approaching eating behaviors). There is likely to be a complex interaction of these factors and future research should aim to explore these mechanisms within prospective studies.

We also found gender differences for several of the appetitive traits. Females were slightly more sensitive to internal satiety mechanisms, tended to eat more slowly, and displayed higher levels of Emotional Over-Eating than males in our study. These results correspond with other adolescent studies showing Emotional Eating to be more evident in females than males (Braet and van Strein, 1997), as well as binge-eating, which is closely related to Emotional Eating (Vereecken et al., 2004; Jones et al., 2010). Adolescent females show more dietary restraint than males (Wardle et al., 1992; Neumark-Sztainer et al., 2007) and theoretical models indicate that Restrained Eating causes increased hunger and thus represents a risk for overeating (Polivy and Herman, 1985), which has been supported with observational research (Stice and Presnell, 2002). This might explain why females displayed more Food Avoidance traits and higher levels of Hunger compared to males. The greater concern about weight and shape reported by adolescent females compared to males (Sweeting and West, 2002), might also contribute to females being more conscious about eating, hunger, and satiety. This might make females more likely to recognize and report internal signals of hunger, as well as aiming for, and thus reporting more food-avoidance. We did not find any associations between maternal/paternal education or ethnicity and any of the appetitive traits. Future research is needed to explore sociodemographic differences further.

Our findings should be interpreted in light of several limitations. Firstly, self-report of appetitive traits could be affected by social desirability, a risk that might have been increased by gathering data in the school setting in the presence of peers (Braet et al., 2007). Adolescents may perceive certain traits as less desirable than others (e.g., Food Responsiveness) and thus under-report these traits, and/or over-report traits considered more favorable (e.g., Satiety Responsiveness). On the other hand, given that social desirability is positively associated with age (Streiner and Norman, 2015), the finding that older participants report higher levels of Food Approach appetitive traits compared to younger age groups does not support such an assumption. Completion of the AEBQ may also have been influenced by other factors, which were not included in this study. For example, we did not include height and weight data that would have enabled us to explore associations between appetitive traits and BMI in this sample. Future studies utilizing the AEBQ with adolescents should explore associations between the AEBQ and adiposity, puberty status or estimated age of puberty onset, physical activity, and the presence or absence of feeding and eating disorders. These factors could have contributed to the better model fit observed for older adolescents, as well as the poor performance of the Hunger subscale in this sample.

Given the model fit of the AEBQ was poorer among 11–13 year olds (with both the CFI and IFI were below 0.9), the results of the MIMIC model should be interpreted carefully. Additional studies are needed to confirm if this is consistently the case, and to understand where, within this age group, the poor model fit arises, and why. Comparisons of the self-report AEBQ with the parent-report CEBQ among 11–13 year olds would be useful, and comparisons of the two measures in adolescents more widely could provide additional evidence for the AEBQ’s validity. Unlike the AEBQ, the parent-report CEBQ has already been validated against behavioral measures (Carnell and Wardle, 2007). Laboratory studies are also needed to examine whether AEBQ scores are reflected in observational measures of eating behavior. Discriminant validity should also be tested for by comparing the AEBQ against other psychometric measures. Future studies should collect more comprehensive sociodemographic information to explore relevant associations and further test the performance of the AEBQ in different sociodemographic groups and culturally divergent samples.

The current research aimed to confirm the factor structure, test internal and test–retest reliability of the AEBQ in adolescents. The results reveal the AEBQ is an appropriate measure of seven appetitive traits in adolescents, with the exclusion of the Hunger subscale, it was internally and externally reliable over time. Food Approach traits were more evident in older compared to younger adolescents, and females displayed more Food Avoidance traits and more Emotional Over-Eating than males. These findings require replication in samples of culturally diverse adolescents. Future studies should also examine the prospective relationship between appetitive traits and explore why young people seem to become more food-approaching with age and the risk this may incur with weight gain.

The scoring system of the AEBQ can be downloaded from the following website: http://www.ucl.ac.uk/iehc/research/behavioural-science-health/resources/questionnaires/eating-behaviour-questionnaires.

## Data Availability

The datasets generated for this study are available on request to the corresponding author.

## Ethics Statement

Ethical approval was granted by University College London Research Ethics Committee. Written informed consent was obtained from all participants over the age of 16 years. Parental consent was obtained for all participants under the age of 16.

## Author Contributions

CH-A contacted the schools, coordinated the data collection, and performed the reliability analyses, linear regressions, and the complex samples general linear models. All authors contributed to the interpretation of data as well as to manuscript preparation. CH-A, HC, CL, and RB contributed to the design of the study. RB supervised the coordination of the study. SS, CH-A, and RB drafted the manuscript. SS performed the confirmatory factor analysis. WG performed the invariance testing and the MIMIC model. All authors read and approved the final manuscript.

## Conflict of Interest

The authors declare that the research was conducted in the absence of any commercial or financial relationships that could be construed as a potential conflict of interest.

## Abbreviations

AEBQ: Adult Eating Behavior Questionnaire
AIC: Akaike’s Information Criteria
BIC: Bayesian Information Criterion
CEBQ: Child Eating Behavior Questionnaire
CFA: Confirmatory Factor Analysis
CFI: comparative fit index
DEBQ: Dutch Eating Behavior Questionnaire
ICC: Intra-class correlation coefficients
IFI: incremental fit index
MIMIC: multiple indicator multiple cause
RMSEA: root mean square error of approximation
TLI: Tucker–Lewis index.
